# Supplementary material for: Multimodal brain age estimates relate to Alzheimer disease biomarkers and cognition in early stages: a cross-sectional observational study
Source: eLife. 2023 Jan 6;12:e81869. doi: 10.7554/eLife.81869 (PMC9988262; doi:10.7554/eLife.81869)
Supplement: Supplementary file 1. — TR = repetition time, TE = echo time. [file elife-81869-supp1.docx]

|  | **TRAINING SETS** | | | **TEST SETS** | | | **ANALYSIS SETS** | | |
| --- | --- | --- | --- | --- | --- | --- | --- | --- | --- |
| **Measure** | **Ances Controls**  **(CN/<50)** | **DIAN Controls**  **(CN/A-)** | **Knight ADRC Controls**  **(CN/A-)** | **Ances Controls**  **(CN/<50)** | **DIAN Controls**  **(CN/A-)** | **Knight ADRC Controls**  **(CN/A-)** | **CN/A-** | **CN/A+** | **CI** |
| **T1 MPRAGE** |  |  |  |  |  |  |  |  |  |
| *Voxel size (mm^3^)* | 1.0 | 1.1 x 1.1 x 1.2 | 1 x 1 x 1.2  or 1.0 | 1.0 | 1.1 x 1.1 x 1.2 | 1 x 1 x 1.2  or 1.0 | 1 x 1 x 1.2  or 1.0 | 1 x 1 x 1.2  or 1.0 | 1 x 1 x 1.2  or 1.0 |
| *TR (ms)* | 2400 | 2300 | 2300 - 2400 | 2400 | 2300 | 2300 - 2400 | 2300 - 2400 | 2300 - 2400 | 2300 - 2400 |
| *TE (ms)* | 3.16 | 2.95 | 2.95 - 3.16 | 3.16 | 2.95 | 2.95 - 3.16 | 2.95 - 3.16 | 2.95 - 3.16 | 2.95 - 3.16 |
| *Flip Angle* | 8° | 9° | 8° - 9° | 8° | 9° | 8° - 9° | 8° - 9° | 8° - 9° | 8° - 9° |
| **Resting-state fMRI** |  |  |  |  |  |  |  |  |  |
| *Voxel size (mm^3^)* | 4.0 | 3.0 – 4.0 | 4.0 | 4.0 | 3.0 – 4.0 | 4.0 | 4.0 | 4.0 | 4.0 |
| *TR (ms)* | 2200 | 2200 – 3000 | 2200 | 2200 | 2200 – 3000 | 2200 | 2200 | 2200 | 2200 |
| *TE (ms)* | 27 | 27-30 | 27 | 27 | 27-30 | 27 | 27 | 27 | 27 |
| *Flip Angle* | 90° | 80° | 90° | 90° | 80° | 90° | 90° | 90° | 90° |
| *# runs / # frames per run* | 2 / 164 | 1 / 120 | 2 / 164 | 2 / 164 | 1 / 120 | 2 / 164 | 2 / 164 | 2 / 164 | 2 / 164 |

**Supplementary File 1.** Summary of acquisition parameters for structural T1 and resting-state functional MRI. TR = repetition time, TE = echo time.
